# Problem formulation by medical students: an observation study

**DOI:** 10.1186/1472-6920-7-16

**Published:** 2007-06-17

**Authors:** Francois Auclair

**Affiliations:** 1 Department of Medicine, University of Ottawa, Canada

## Abstract

**Background:**

Medical problems are often complex and ill-structured. In formulating the problem, one has to discriminate pertinent elements from irrelevant information in order to effectively find a solution. In this observation study, we describe how medical students formulate the problem of a complex case.

**Methods:**

32 third year medical students were presented with a complex case of endocarditis. They were asked to synthesize the case and give the best formulation of the problem. They were then asked to provide a diagnosis. A subsequent group of 25 students were presented with the problem already formulated and were also asked for the diagnosis. We analyzed the student's problem formulations using the presence or absence of essential elements of the case, the use of higher-order concepts and the use of relations between concepts.

**Results:**

12/32 students presented with the case made the correct diagnosis. Diagnostic accuracy was significantly associated with the use of higher-order concepts and relations between concepts. Establishing explicit relations was particularly important. Almost all students who missed the diagnosis could not elicit any relations between concepts but only reported factual observations. When presented with an already formulated problem, 19/25 students made the correct diagnosis. (p < 0.05)

**Conclusion:**

When faced with a complex new case, students may not have the structured knowledge to recognize the nature of the problem. They have to build new schema or problem representation. Our observations suggest that this process involves using higher-order concepts and establishing new relations between concepts. The fact that students could recognize the disease when presented with a formulated problem but had more difficulty when presented with the original complex case indicates that knowledge of the clinical features may be necessary but not sufficient for problem formulation. Our hypothesis is that problem formulation represents a distinct ability.

## Background

Problem formulation is necessary because the world we experience is complex. The problems we face are often ill-structured. One or several elements of the problem may be unknown, the same elements may be different in different context, there is uncertainty about the concepts necessary for a solution, the relationship between concepts and rules may be inconsistent between cases. We need to organize the information into sensible patterns [1].

We can define problem formulation as a structure of concepts linked by relations. The role of such a system is to organize the problematic experience in such a way that enables better understanding of the state of affairs so that we can more effectively search for solutions [2,3]. As stated by Rosenhead [4]: "the most demanding and troubling task in formative decision situations is to decide what the problem is".

Problem formulation is not an objective procedure. It is a creation that is probably dependent on norms, values, knowledge, perception of the situation, problem environment and past experience [2,5]. In medicine, problem formulation is a created synthesis that ideally would contain only those attributes that are deemed significant [6]. It has been well documented that integration of irrelevant information from past experience may lead to inaccurate diagnosis [7].

In this study, we investigated how medical students formulate the problem of a complex medical case. We analyzed the characteristics of their formulated problems and examined if there was any correlation with diagnostic accuracy. We also tested the hypothesis that problem formulation is a creative ability in which knowledge of signs and symptoms of a disease may be necessary but insufficient for making a diagnosis.

## Methods

Seven small groups of third year medical students (total 32 students) doing their Internal Medicine rotation were successively presented with a complex case of endocarditis from the New England Journal of Medicine [8]. This was done in the context of clinical reasoning sessions in which students are presented with an unknown case followed by interactive discussion on solving the problem. The dual purpose was to learn about a specific infectious disease and foster reflective practice. Students were instructed to regroup the clinical information into the smallest number of concepts that represent the whole case and to write it down as their best possible formulation of the problem. They were given a written example of what was expected. They were given all the time required to complete the task. At the end, they were asked for their diagnosis. This was followed by interactive discussion that included teaching about the disease and resolution of the case at the end. The duration of the session was two hours.

We considered five elements or observations to be essential to the case namely: the occurrence of pneumonia, the positive blood cultures with Streptococcus pneumoniae, the complication of endophthalmitis, the presence of a heart murmur and the development of hemodynamic problems. These were used for presenting a formulated problem. The formulated problem was constructed according to accepted criteria for the clinical diagnosis of endocarditis [9].

We analyzed the students' problem formulation with the following characteristics: the presence or absence of the above essential elements, the use or not of higher-order concepts (or new conceptual category) and the mention or not of relations between concepts. For higher-order concepts we considered any introduction of a new conceptual category from the observation terms. These included semantic qualifier as described by Bordage [10] in which the content of an observation is given an abstract form along oppositional relationships. For example, pneumonia could be qualified as acute or chronic. We also included any abstraction into a larger set such as a generalization from multiple terms. For example, the combination of leucopenia and use of corticosteroids could be subsumed under 'immunocompromised'.

Relations we looked for were related to causation. We look for direct expression of causation in which the student would state that an element brings about or progress into another one or that an element was an effect of another. We also looked for temporal relations of continuity or succession in which student would state that an event follows or is preceded by another. These are not necessarily causal but often associated with causation. We examined if there was any significant association between the characteristics of problem formulation and diagnostic accuracy using Fisher's exact test.

In order to examine the role of previous knowledge, we presented a formulated problem of the same case of endocarditis to five subsequent groups of third year students (total 25 students) also doing their rotation in Internal Medicine. The students were asked to make a diagnosis based on the following formulated problem: "A 43 year old man presents with a history of complicated pneumococcal pneumonia with empyema, positive blood cultures, and subsequent development of left eye endophthalmitis, heart murmur and sudden onset of heart failure requiring intubation". The problem was used as an illustration of the importance of problem formulation and was followed by different cases presentations.

The study was reviewed and approved by the Education Research committee of the Faculty of Medicine of the University of Ottawa. No further ethical approval was requested as the primary focus of these sessions is teaching. The clinical reasoning sessions are part of the regular curriculum for third year medical students. Our study is a report on observations made during teaching sessions. The collection of data was made with the informed consent of participating students and participation to the sessions was voluntary.

## Results

12/32 students presented with the case made a correct diagnosis of endocarditis.

We observed significant differences in the nature of problem formulation between students. Students with the correct diagnosis were more likely to use higher-order concepts and to make relations between concepts explicit than those with incorrect diagnosis (p < 0.05) (Tables 1). There was no difference in the frequency of including any of the five essential elements between students who had the right diagnosis and those who did not (Table 2).

**Table 1:** Use of higher-order concepts and relations between concepts by students and diagnostic accuracy

	Correct diagnosis	Wrong diagnosis	p-value
Students using higher-order concepts	11/12 (92%)	11/20 (55%)	0.03
Students making relations between concepts	6/12 (50%)	1/20 (5%)	0.005

**Table 2:** Identification of essential clinical features and diagnostic accuracy

Clinical feature identified	Correct diagnosis	Wrong diagnosis	p-value
Pneumonia	12/12 (100%)	19/20 (95%)	0.62
Bacteremia	6/12 (50%)	12/20 (60%)	0.24
Endophthalmitis	12/12 (100%)	19/20 (95%)	0.62
Heart murmur	4/12 (33%)	3/20 (15%)	0.16
Hemodynamic problem	10/12 (83%)	16/20 (80%)	0.35

The role of relations between concepts was particularly revealing. Although the students who missed the diagnosis elicited the same number of relevant clinical findings as those who made the diagnosis, they were unlikely to make explicit any relation between concepts (1/20).

The group of students presented with a formulated case seemed to have sufficient knowledge to recognize the disease. When first presented with a synthesis of the case, a high proportion of those students were able to make an accurate diagnosis (19/25) by comparison to the groups of students presented with the original complex case (12/32) (p < 0.05).

## Discussion

Our first set of observations suggests that the use of higher-order concepts and making explicit relations between concepts are associated with diagnostic accuracy. Bordage and Chang [10,11] have demonstrated that diagnostic accuracy correlates with semantic level of the problem's description. The use of semantic qualifiers that describe the content at a more abstract level was associated with better diagnostic accuracy. Abstractions from observation concepts constitute interpretations. Those interpretations confer additional meaning [12]. Qualifying pneumonia as recurrent or persistent is an interpretation of a series of events and adds to the meaning of the concept of pneumonia and may thus increase diagnostic accuracy.

In another experiment, Nendaz and Bordage [13] instructed students how to use more semantic qualifiers. They found that students could learn to introduce more semantic qualifiers but there was no difference in diagnostic accuracy suggesting that the use of semantic qualifiers correlates with diagnosis but may not be causal.

Our observations may provide a possible explanation to this apparent lack of causation between use of semantic qualifiers and diagnostic accuracy. We found that relations between concepts need to be established. Absence of explicit relations between concepts in problem formulation was associated with incorrect diagnosis. The use of conceptual abstraction may be necessary but not sufficient for problem formulation; a critical element is the structure resulting from relations established between concepts. Establishing meaningful relations has also been shown by Norman [14] to be important in problem solving. Physicians with different levels of experience were presented with complex nephrology problems and asked to solve them while thinking aloud. Experienced physicians solved the problems by clustering data into more meaningful relations than less experienced ones. We have observed that conceptual relations need to be established early in the problem formulation.

Why would establishing relations between concepts be associated with better diagnostic accuracy? Our observations suggest that the structure of problem formulation is analogous to that of a model of a theory [15]. In the semantic view of theory, a model can be a linguistic entity on which observation concepts are abstracted into theoretical terms. Those are organized in a structure which contains as minimal requirements: concepts and a set of relations or operations on those concepts. The relations are made explicit. With such a structure the model may represent the world and have an explanatory function [16]. Like a model, a problem formulation can make explicit functional and causal relations between concepts. A model of a case of endocarditis will link bacteremia, valvular disease and embolic phenomena in causal relationships and these have explanatory utility. The formulated problem will allow the physician to see the case as belonging to a 'theory' of endocarditis. What is shared between the model and the theory is not only a set of features of individual concepts but the same pattern of abstract relationships. Choosing the pertinent concepts and establishing relations most likely involve analytic and non-analytic processes [17]. Non-analytical recognition of similar cases from past experience has been associated with expertise [18] and likely involves seeing relations between concepts. On the other hand, analysis of specific features, weighting prior probability, and consideration for simplicity must also play a role in formulating the problem.

In the second set of observations, we found that a majority of students could readily recognize the disease when presented with an already formulated problem. This was in contrast to the groups of students presented with the original case. A possible explanation would be that students who made the diagnosis had already structured knowledge of endocarditis. Why those groups would have such a structured knowledge is unexpected since there was no new course or teaching on the subject in those groups. Moreover, several preceding groups had significant difficulty in making the diagnosis.

More likely, the explanation would be that when presented with the original complex case, the difficulty was in structuring the elements of the problem into a recognizable form. Very few students identified the regurgitant murmur as an essential feature. Possibly, when presented with the formulated case in which the murmur is mentioned, students recognized the disease as endocarditis. Faced with the large amount of clinical data of the original case, students may have had problem seeing relations between pertinent clinical features. Medin [19] has shown that when relationships between properties were exhibited, particularly causal relationships, subject were better able to assign objects to a similar category.

There are several limitations to our study. These were observations made during teaching sessions. The groups of students were not randomized and were seen in sequence with the last five groups presented with the formulated case. It is possible that students who made the diagnosis had different prior experiences with similar cases or different knowledge structures. This should not invalidate the observations on the structure of problem formulation but would cast doubt on our hypothesis that problem formulation is a creative ability. The formulated problem and the criteria for higher-order concepts and what constitutes relations between concepts were not validated by other physicians. The formulated problem was based on accepted clinical criteria of endocarditis and unlikely would cause significant dissension among experts. The choice of higher-order concepts and relations would be subject to interpretation. However, the interpretation would concern more the type of relations or concepts than whether a relation is made explicit or not or whether a new conceptual category is introduced or not. No observational term was accepted as new concept. This study was also limited to the use of one complex case. Conceptual analysis with abstractions and relations may not be so important in other cases. In many instances, the single ability to detect critical features may be more important. These observations may not apply to less complex case. No doubt, there are many ways to adequately formulate a problem.

## Conclusion

With practice, experts develop schema or scripts that enable them to recognize a situation as belonging to a certain class of problem [20,21]. Such schema can make sense of new complex information and consist of structured knowledge. There is now evidence that acquiring multiple representations of knowledge is more important in clinical reasoning than any particular strategy such as hypothetico-deductive reasoning [17,22]. In novel situations, schema may not be directly available for searching the relevant elements of the problem and one must build a new problem representation [1]. This process involves creative thinking. Cognitive and psychometric approaches in the study of creativity suggest that mental representations are involved in new combinations with processes like association, synthesis, analogical transfer or categorical reduction where elements are reduced to more primitive descriptions [23]. Our observations suggest that problem formulation involves not only abstraction but also making new relations between concepts and that it may be a creative ability for which knowledge of clinical features of a disease may be necessary but not sufficient.

## Competing interests

The author(s) declare that they have no competing interests.

## Authors' contributions

The author has developed the clinical reasoning sessions in Infectious Diseases and has analysed the structure of the problems formulated by the medical students.

## Pre-publication history

The pre-publication history for this paper can be accessed here:


